# Resistance exercise training with protein supplementation improves skeletal muscle strength and improves quality of life in late adolescents and young adults with Barth syndrome: A pilot study

**DOI:** 10.1002/jmd2.12244

**Published:** 2021-08-09

**Authors:** Kathryn L. Bohnert, Grace Ditzenberger, Adam J. Bittel, Lisa de las Fuentes, Manuela Corti, Christina A. Pacak, Carolyn Taylor, Barry J. Byrne, Dominic N. Reeds, W. Todd Cade

**Affiliations:** ^1^ Program in Physical Therapy Washington University School of Medicine St. Louis Missouri USA; ^2^ Doctor of Physical Therapy Division Duke University School of Medicine Durham North Carolina USA; ^3^ Department of Medicine Washington University School of Medicine St. Louis Missouri USA; ^4^ Department of Pediatrics University of Florida School of Medicine Gainesville Florida USA; ^5^ Department of Pediatrics Medical University of South Carolina Chaleston South Carolina USA; ^6^ Center for Human Nutrition Washington University School of Medicine St. Louis Missouri USA

**Keywords:** amino acid, exercise, muscle, strength training

## Abstract

**Background:**

Muscle weakness and exercise intolerance contribute to reduced quality of life (QOL) in Barth syndrome (BTHS). Our group previously found that 12 weeks of resistance exercise training (RET) improved muscle strength, however, did not increase muscle (lean) mass or QOL in n = 3 young adults with BTHS. The overall objective of this pilot study was to examine the safety and effectiveness of RET plus daily protein supplementation (RET + protein) on muscle strength, skeletal muscle mass, exercise tolerance, cardiac function, and QOL in late adolescents/young adults with BTHS.

**Methods:**

Participants with BTHS (n = 5, age 27 ± 7) performed 12 weeks of supervised RET (60 minutes per session, three sessions/week) and consumed 42 g/day of whey protein. Muscle strength, muscle mass, exercise capacity, cardiac function, and health‐related QOL were assessed pre‐post intervention.

**Results:**

RET + protein was safe, increased muscle strength and quality of life, and tended to increase lean mass.

**Conclusions:**

RET + protein appears safe, increases muscle strength and quality of life and tends to increase lean mass. Larger studies are needed to confirm these findings and to fully determine the effects of RET + protein in individuals with BTHS.

## 1 INTRODUCTION

Barth Syndrome (BTHS) is a rare, X‐linked disorder caused by a mutation of the tafazzin gene (*TAFAZZIN*), which is responsible for the remodeling of the phospholipid cardiolipin in the mitochondrial membrane.^1^ Cardiolipin plays an essential role in maintaining the structure of the mitochondrial membrane and stabilizes the respiratory supercomplexes to facilitate mitochondrial energy production.^2^, ^3^ Subsequently, the pathological remodeling of cardiolipin manifests in heart failure, arrhythmia, exercise intolerance, and abnormal fatty acid and glucose metabolism.^1^, ^4^, ^5^, ^6^

A key contributor to exercise intolerance is muscle weakness, which in BTHS is typically first manifested as failure to thrive and delayed achievement of motor milestones early in life.^1^ Our group^7^ and others^8^ further demonstrated that skeletal muscle strength in school‐age children, adolescents, and young adults with BTHS is approximately 50% of that of age‐matched peers. Skeletal muscle weakness might be especially impactful in BTHS as evidenced by a recent FDA Externally‐Led Patient Focused Drug Development Meeting where 86% of participants with BTHS reported that skeletal muscle weakness/exercise intolerance had greater impact on their lives than any other symptom including heart failure (37%), dysrhythmias (13%), and neutropenia (54%).^9^ This suggests that interventions to improve skeletal myopathy/muscle weakness would be particularly beneficial in people living with BTHS.

Our group has recently reported, in a small pilot study, that 12 weeks of supervised resistance exercise training (RET) improved muscle strength in young adults with BTHS.^7^ Albeit a small sample, muscle strength increased but there was no concomitant increase in skeletal muscle mass. We proposed that this lack of improvement in muscle mass might have been due to higher amino acid turnover in BTHS^10^, ^11^ and that provision of additional daily protein, when combined with RET, may improve both strength and skeletal muscle mass.

Given the apparent anabolic resistance seen in our pilot study, the primary objective of the pilot study was to collect preliminary data on the safety and efficacy of resistance training combined with daily supplemental protein (whey protein isolate ‐ 42 g/day) on muscle strength and mass, exercise tolerance, heart function, and quality of life in a late adolescents and young adults with BTHS. We hypothesized that RET plus supplemental protein would increase skeletal muscle strength and mass and improve the quality of life subdomains associated with physical activity/fatigue.

## 2 METHODS

### 2.1 Participants

Five (n = 5, age 27 ± 7), sedentary (no routine exercise ≥2x/week) male participants with BTHS were recruited from the Barth Syndrome Foundation Registry located at the University of Florida and entered this 12‐week study. Medications used by participants included beta blockers (n = 5, carvedilol, atenolol), ACE inhibitors/angiotensin receptor blockers (n = 3, lisinopril and losartan), cardiac glycosides (n = 4, digoxin), and granulotcyte colony stimulating factor (n = 2, neupogen). Participants (n = 4, did not have mutation data on n = 1) had the following *TAFAZZIN* mutations: exon 2: frameshift deletion, exon 10: nonsense, exon 2: nonsense, and exon 2: splicing defect. Baseline and follow‐up testing were performed at the Washington University Institute for Clinical and Translational Sciences (ICTS) Clinical Research Unit and included a medical history and physical, body composition analysis, and fasting blood chemistries including a complete blood count (CBC) and comprehensive metabolic panel (CMP). Postintervention testing occurred 48‐72 hours after the final exercise session. Studies were approved by the Human Studies Committee at Washington University in St. Louis and all participants and parents (ie, adolescents) provided written informed consent.

### 2.2 Body composition

Body composition measurements were performed in n = 4 participants using dual‐energy X‐ray absorptiometry (DXA) (Hologic Discovery W QDR Series, Malborough, Massachusetts). Regional and whole body fat, lean mass, bone mineral content, and bone mineral density were assessed. A certified technician performed both pretraining and posttraining tests. Body composition was performed in one participant by using air displacement plethymosgraphy (BodPod, LifeMeasurements Inc., Concord, California) due to technical difficulties with DXA at the time of the baseline visit.

### 2.3 Echocardiography

Two‐dimensional (2D), M‐mode, pulsed‐wave Doppler, tissue Doppler echocardiography, and 2D speckle‐tracking global longitudinal strain was performed on all participants (GE Healthcare Vivid E9; Waukesha, Wisconsin) as previously described (Bashir et al.^12^).

### 2.4 Muscle strength and function

One‐repetition weight maximum (1‐RM): 1‐RM is the maximal weight a participant can lift, through the full range of motion, just once using proper form and not substituting other groups of muscles to complete the lifting motion. 1‐RM weight was recorded for the leg press, bench press, biceps curl, seated row, knee extension, and shoulder press according to guidelines established by the American College of Sports Medicine (2000) by using the Hoist Fitness Systems (H2200; San Diego, California) at both pretraining and posttraining testing supervised by a trained research team member.

### 2.5 Quality of life

Participants completed the Minnesota Living with Heart Failure Questionnaire (MLWHFQ), a 21‐item survey examining the effects of heart failure on their QOL (Rector and Cohn^13^). Domains included physical, social, and emotional well‐being. Responses are graded on a 6‐point Likert scale from 0 (having no effect on QOL) to 5 (very much affecting QOL), in which a lower total score represents a better quality of life.

### 2.6 Exercise tolerance testing

Exercise tolerance was assessed before and after the intervention by performing a graded exercise test using a ramped protocol on a recumbent cycle ergometer (Lode, The Netherlands). Exercise intensity (ie, work rate) was increased by 10 W/min while cycling at 60 rpm until volitional exhaustion was reached. 12‐lead ECG, blood pressure, ratings of perceived exertion, oxygen consumption (VO_2_), carbon dioxide production (VCO_2_), ventilation (VE), and respiratory exchange ratio (RER) (ParvoMedics, Sandy, Utah) were continuously collected during the test. Achievement of peak exercise was determined by attainment of ≥85% predicted peak heart rate (220 − age) and/or RER ≥ 1.10 according to the American College of Sports Medicine.^14^

### 2.7 Resistance exercise training

All subjects participated in a 12‐week, supervised, progressive RET regimen performed at a physical therapy or cardiac rehabilitation clinic local to the participant's home. Participants trained 3x/week for 60 minutes at 60% 1 RM for the first 18 sessions, with the intensity increased to 70% 1 RM for the last 18 sessions as tolerated. All participants were trained to complete a 3‐seconds concentric, and 3‐seconds eccentric lifting cadence, and performed three sets of 6 to 10 repetitions per set with 2 minutes of rest between sets for eight lifts: knee extension, knee flexion, leg press, ankle plantar flexion, chest press, seated row, biceps curl, and overhead press. 1‐RM for each participant was retested every 10 sessions, with the weight on each lift adjusted to maintain the prescribed intensity. Training sessions were supervised by a licensed physical therapist or exercise physiologist, who monitored participants' heart rate, blood pressure, and levels of perceived exertion throughout training.

### 2.8 Protein supplementation

Participants consumed 42 g/day of whey protein isolate in the form of whey protein powder and/or protein bars (UNJURY, Sterling, Virginia). The protein powder could be added to liquid (flavored) or sprinkled over food (unflavored). Each protein bar had approximately 21 g of protein. The amino acid composition of the protein powder and bars are provided in Table 1. On training days, participants were instructed to consume a protein shake or bar within 1 hour of completing the resistance exercise session as the effects of protein supplementation with resistance exercise appears to be most effective if taken within 2 hours postexercise.^15^

**TABLE 1:** Amino acid composition in protein supplements

UNJURY protein powder	Percent amino acid (%)
Alanine	4.93
Arginine	2.08
Aspartic acid	10.55
Cystine	2.41
Glutamic acid	17.01
Glycine	1.60
Histidine	1.58
Isoleucine	6.40
Leucine	10.37
Lysine	10.07
Methionine	2.03
Phenylalanine	2.91
Proline	5.91
Serine	4.59
Threonine	6.76
Tryptophan	2.37
Tyrosine	2.80
Valine	5.63
	100.00
AA Profile UNJURY brownie bars	
Alanine	2.95
Arginine	3.22
Aspartic acid	8.63
Cystine	0.85
Glutamic acid	19.18
Glycine	1.80
Histidine	2.40
Isoleucine	5.40
Leucine	9.19
Lysine	7.57
Methionine	2.62
Phenylalanine	4.43
Proline	8.95
Serine	5.90
Threonine	4.91
Tryptophan	1.34
Tyrosine	4.69
Valine	6.08
	100.10
AA Profile UNJURY chocolate peanut butter bars	
Alanine	5.03
Arginine	2.01
Aspartic acid	10.85
Cystine	2.13
Glutamic acid	17.45
Glycine	1.57
Histidine	1.68
Isoleucine	6.71
Leucine	10.51
Lysine	9.17
Methionine	2.13
Phenylalanine	2.91
Proline	5.70
Serine	4.36
Threonine	7.05
Tryptophan	1.90
Tyrosine	2.80
Valine	6.04
	100.00

### 2.9 Statistical analysis

Differences between pretesting and posttesting for all outcomes were determined using Wilcoxon Rank Sum test. Significance was determined at *P* ≤ .05 (IBM SPSS Statistics for Windows, version 27 [IBM Corp., Armonk, New York]).

## 3 RESULTS

### 3.1 Dietary intake, safety of RET + protein, and plasma metabolic, cardiac, and immune function

Participant (n = 4, one participant did not return diary) dietary intake including total calories (pre: 2512 ± 1162 vs post: 2290 ± 743 kcal, *P* = .76), total protein (pre: 84.0 ± 30.6 vs post: 72.7 ± 23.2 g, *P* = .58), total carbohydrate (pre: 330.6 ± 166.5 vs post: 334.7 ± 167.4 g, *P* = .97), and total fat (pre: 94.9 ± 49.7 vs post: 73.2 ± 26.1 g, *P* = .47) did not change pre‐post intervention. As per participant report, compliance with the supplemental protein intake was >90%. The intervention was well tolerated among participants with no adverse events or complications. Plasma concentrations of glycine and glutamic acid tended (*P* = .07) to increase following the intervention (Table 2). Biomarkers of kidney function (plasma creatinine), myocardial damage (creatine kinase‐myocardial band), and heart failure (brain natriuretic peptide) did not change after the intervention (Table 3). There was no change in hematologic values, glucose or plasma lipid profiles (Table 3).

**TABLE 2:** Impact of 12 weeks RET + protein on plasma amino acid concentrations

Measure	Pretraining	Posttraining	*P* value
Phenylalanine	80.0 (9.1); 79 [72‐89]	70.5 (7.9); 69 [64‐78]	.14
Tyrosine	81.5 (22.3); 86 [59‐100]	80.3 (14.4); 86 [65‐90]	.72
Isoleucine	75.3 (15.3); 79 [59‐88]	68.8 (16.5); 73 [52‐82]	.47
Leucine	141.0 (19.8); 146 [121‐157]	147.5 (27.8); 158 [118‐167]	.47
Valine	307.0 (72.6); 310 [235‐376]	264.5 (62.6); 283 [199‐312]	.14
Alloisoleucine	1.5 (1.0); 2 [0.5‐2]	1.0 (0.8); 1 [0.3‐2]	.16
Threonine	315.5 (93.1); 292 [241‐414]	290.5 (83.3); 292 [213‐366]	.72
Serine	153.5 (65.2); 129 [110‐222]	157.0 (34.8); 165 [121‐186]	1.00
Glycine	221.0 (113.7); 179 [145‐340]	288.8 (132.0); 288 [165‐414]	.07
Methionine	34.0 (4.2); 33 [31‐39]	47.0 (25.4); 35 [33‐73]	.47
Homocysteine	0.0 (0.0); 0 [0–0]	0.0 (0.0); 0 [0‐0]	1.00
Cystathionine	0.0 (0.0); 0 [0–0]	0.0 (0.0); 0 [0‐0]	1.00
Cystine	19.3 (9.3); 18 [12‐29]	19.3 (3.0); 19 [17‐22]	1.00
Glutamine	563.0 (77.1); 548 [500‐641]	615.5 (185.5); 549 [486‐813]	.41
Glutamic acid	128.0 (15.8); 133 [111‐140]	184.5 (48.9); 169 [148‐237]	.07
Citrulline	36.5 (36.6); 22 [14‐74]	31.5 (25.8); 21 [16‐58]	1.00
Argininosuccinic acid	0.0 (0.0); 0 [0–0]	0.0 (0.0); 0 [0‐0]	1.00
Arginine	76.0 (5.0); 76 [72‐81]	70.5 (33.7); 62 [44‐106]	.72
Ornithine	64.3 (27.5); 62 [40‐91]	94.0 (70.0); 94 [28‐161]	.27
Homocitrulline	0.3 (0.5); 0 [0‐0.8]	0.5 (0.6); 0.5 [0–1]	.32
Alanine	399.0 (46.2); 417 [351‐430]	370.3 (72.8); 380 [296‐435]	.27
Hydroxyproline	15.3 (3.9); 16 [11‐19]	23.8 (10.5); 27 [13‐32]	.14
Proline	280.3 (49.6); 290 [230‐322]	258.8 (70.5); 248 [198‐331]	.47
Lysine	178.3 (21.6); 176 [160‐200]	199.8 (52.0); 191 [156‐253]	.29
Alpha‐aminoadipic acid	1.5 (1.3); 1.5 [0.3‐2.8]	2.3 (1.9); 1.5 [10–4]	.18
Beta‐aminoisobutyric acid	1.8 (1.5); 1 [1–3]	1.8 (1.5); 1 [1–3]	1.00
Beta‐alanine	6.5 (2.4); 5.5 [5–9]	7.8 (2.2); 8 [6‐10]	.29
Sarcosine	2.5 (1.3); 2.5 [1–4]	3.3 (1.0); 3.5 [2–4]	.28
Gamma‐aminobutyric acid	0.3 (0.5); 0 [0‐0.8]	0.3 (0.5); 0 [0‐0.8]	1.00
Histidine	77.3 (22.5); 70 [62‐100]	108.3 (26.4); 109 [83‐133]	.07
Carnosine	0.0 (0.0); 0 [0–0]	0.3 (0.5); 0 [0‐1]	.32
Alpha‐aminobutyric acid	43.8 (24.0); 47 [19‐65]	41.8 (22.9); 45 [19‐62]	.72

*Note*: Values are mean (SD) and median [IQR].

**TABLE 3:** Effects of 12 weeks RET + protein on plasma metabolic, muscle, and immune biomarkers

Measure	Pretraining	Posttraining	*P* value
Prealbumin (mg/dL)	18.7 (5.1); 18 [14‐24]	17.9 (5.2); 18 [13‐23]	.50
Glucose (mg/dL)	106.4 (31.4); 101 [83–133]	108.4 (23.4); 107 [89‐129]	.89
Triglycerides (mg/dL)	103.2 (33.8); 92 [74‐139]	98.2 (74.4); 66 [55‐158]	.50
Total cholesterol	116.8 (16.7); 111 [102‐135]	120.2 (37.9); 106 [96‐152]	.50
Neutrophils (%)	42.9 (13.6); 44 [29‐56]	45.8 (16.3); 39 [32‐63]	.69
ANC (cells/mm^3^)	1.9 (0.9); 2.2 [1‐2.6]	2.1 (0.8); 2.6 [1.1‐2.7]	.47
Creatinine (mg/dL)	0.6 (0.1); 0.6 [0.5‐0.7]	0.7 (0.2); 0.6 [0.5‐0.9]	.35
Hemoglobin (g/dL)	13.9 (1.5); 13 [13‐15]	14.0 (1.9); 13 [13‐16]	1.00
HCT (%)	42.2 (5.1); 40 [38‐47]	42.3 (5.2); 39 [38‐48]	.89
AST (units/L)	28.4 (5.1); 27 [25‐33]	30.4 (4.6); 30 [26‐35]	.35
ALT (units/L)	29.4 (6.7); 27 [26–35]	29.2 (6.5); 31 [23‐35]	.89
CK‐MB (% of total CK)	2.6 (0.6); 3 [2–3]	4.7 (1.5); 5 [3–5]	.11
BNP (pg/mL)	55.4 (45.7); 66 [8‐98]	291.2 (466.5); 75 [32‐660]	.50

*Note*: Values are mean (SD) and median [IQR].

Abbreviations: ANC, absolute neutrophil count; ASP, aspartate aminotransferase; ALT, alanine aminotransferase; BNP, brain natriuretic peptide; CK‐MB, creatine kinase‐myocardial band; HCT: hematocrit.

### 3.2 Muscle strength and function with resistance exercise training

**FIGURE 1:**
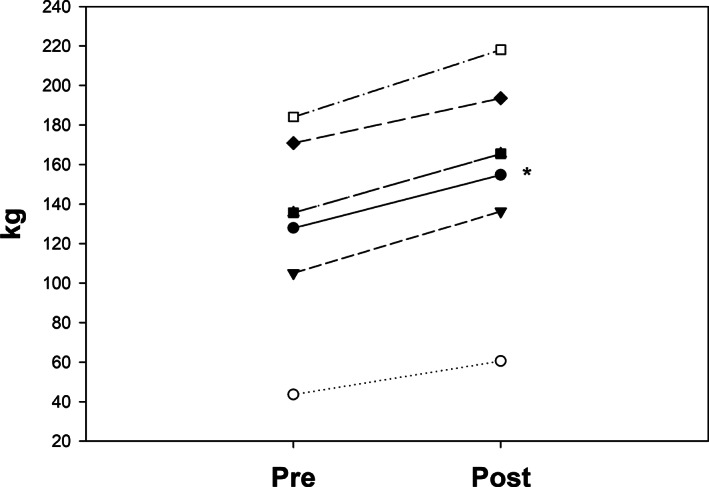
One repetition maximum (1 RM) of combined exercises (kg) pre‐and post‐RET + protein supplementation. Solid line: mean of all participants. *P < .001

Total weight for all participants (ie, all sets and repetitions for all exercises combined) lifted from session 1 to session 36 increased from 4206 ± 1776 kg to 7074 ± 2420 kg (*P* < .03). Muscle strength increased for leg press, bench press, seated row, and shoulder press on 1‐RM testing (all *P* < .05) and tended to increase muscle strength for biceps curl (Table 4). Skeletal muscle strength expressed as a total score of 1‐RM increased following RET + protein (Figure 1).

**TABLE 4:** Effects of 12 weeks of RET + protein on skeletal muscle mass, strength, bone mineral density, exercise tolerance, and cardiac function

Measure	Pretraining	Posttraining	*P* value
Body composition			
Arm lean (kg)	2.4 (0.4); 2.4 [2.0‐2.7]	2.4 (0.3); 2.4 [2.1‐2.7]	.07
Leg lean (kg)	7.3 (1.2); 7.6 [6.0‐8.1]	7.4 (1.1); 7.7 [6.2‐8.2]	.11
Trunk lean (kg)	23.3 (3.4); 23.9 [19.9‐26.3]	24.0 (3.6); 24.6 [20.2‐27.1]	.07
Whole body lean (kg)	45.8 (6.6); 47.4 [38.9‐51.1]	46.9 (6.6); 48.2 [40.0‐52.4]	.07
Whole body fat (kg)	22.2 (10); 25.5 [11.1‐30.0]	22.0 (9.5); 25.6 [12.0‐28.5]	.72
Total mass (kg)	69.9 (16); 75.7 [53.3‐80.7]	70.8 (15); 76.3 [54.7‐81.2]	.47
Skeletal muscle index (%)	33.25 (4.7); 32.4 [29.3‐38.1]	33.42 (4.6); 32.0 [30.1‐38.2]	.72
Arm BMD (g/cm^2^)	0.67 (0.10); 0.67 [0.6‐0.7]	0.67 (0.10); 0.7 [0.6‐0.7]	.41
Leg BMD (g/cm^2^)	0.98 (0.10); 0.97 [0.9‐1.1]	0.97 (0.10); 0.96 [0.9‐1.1]	.28
Thoracic spine BMD (g/cm^2^)	0.72 (0.10); 0.69 [0.7‐0.8]	0.79 (0.10); 0.8 [0.7‐0.9]	.47
Lumbar spine BMD (g/cm^2^)	0.91 (0.10); 0.89 [0.8‐1.0]	0.93 (0.10); 0.9 [0.9‐1.0]	.26
Pelvic BMD (g/cm^2^)	0.98 (0.10); 0.96 [0.9–1.1]	0.98 (0.10); 0.97 [0.9‐1.0]	.56
Exercise tolerance			
VO_2peak_ (mL/kg/min)	12.5 (2.1); 11.5 [11.1‐14.5]	12.0 (2.1); 11.8 [9.9‐14.1]	.28
Max HR (bpm)	156 (11); 153 [146‐167]	146 (17); 151 [129‐162]	.04
Max ventilation (L/min)	42.1 (16.3); 39.4 [28‐58]	41.6 (14.7); 42.4 [29‐54]	.69
Max RER	1.4 (0.2); 1.4 [1.3‐1.6]	1.4 (0.1); 1.5 [1.4‐1.5]	.69
Cardiac function			
Resting HR (bpm)	81 (8); 79 [74‐89]	79 (9); 82 [72‐85]	.50
Resting SBP (mmHg)	106 (11); 104 [98‐114]	95 (7); 99 [88‐101]	.04
Resting DBP (mmHg)	67 (7); 70 [61‐73]	58 (6); 60 [52‐64]	.04
LVM 2D	136.4 (40.5); 140 [117‐167]	119.6 (36); 170 [116‐231]	.27
Ejection fraction (%)	58 (8); 60 [51‐65]	56 (9); 60 [47‐65]	.71
Global strain (%)	−16 (2); −16.1 [−17.9‐0]	−15 (3); −13.9 [−16‐0]	.10
Muscle strength			
Leg press 1 RM (kg)	52 (21); 132 [69‐154]	59 (23); 154 [80‐166]	.07
Knee extension 1 RM (kg)	19 (10); 48 [21‐60]	23 (11); 53 [28‐70]	.11
Biceps curl 1 RM (kg)	9 (5.4); 17.5 [8.8‐33]	12 (5.9); 30 [14‐39]	.07
Seated row 1 RM (kg)	18 (8.6); 45 [21‐56]	23 (7.7); 51 [33‐65]	.04
Bench press 1 RM (kg)	16 (5.4); 40 [24‐45]	22 (7.7); 45 [33‐64]	.04
Shoulder press 1 RM (kg)	13 (7.3); 24 [16‐44]	17 (7.3); 34.5 [24‐53]	.04
Total 1 RM (kg)	127 (56); 298.5 [164‐391]	155 (61); 364 [217‐453]	.04
Quality of life			
MNLWHF total score	44 (40); 33 [10.5‐82]	28 (40); 4.0 [0.5‐67]	.04

*Note*: Values are mean ± SD and median [IQR].

Abbreviations: BMD, bone mineral density; DBP, diastolic blood pressure; HR, heart rate; LVM 2D, left ventricular mass measured by two‐dimensional echocardiography; MNLWHF, Minnesota Living with Heart Failure questionnaire; RER, respiratory exchange ratio; SBP, systolic blood pressure; 1 RM, one repetition maximum.

### 3.3 Body composition and bone mineral density

**FIGURE 2:**
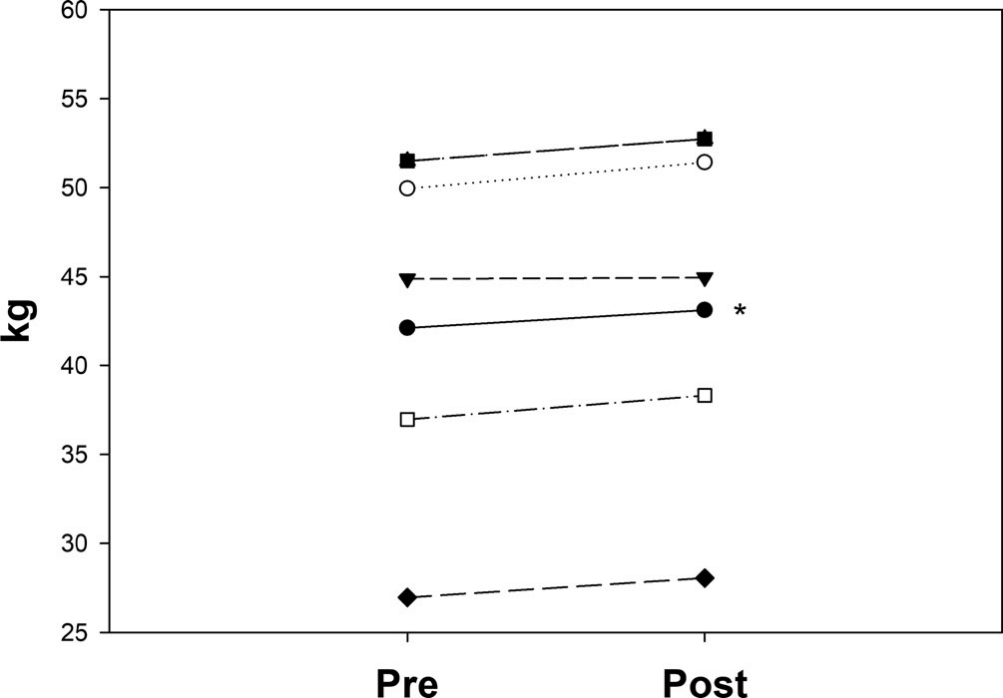
Lean mass (kg) pre‐and post‐RET + protein supplementation. Solid line: mean of all participants. *P < .07

Whole‐body lean muscle mass tended to increase with RET + protein (Figure 2 and Table 4). Spine, appendicular or pelvic bone mineral density did not change following RET + protein (Table 4).

### 3.4 Cardiac function and exercise tolerance

Systolic and diastolic blood pressure decreased following RET + protein (Table 4). Echocardiographic measures of left ventricular mass and systolic function did not change following RET + protein (Table 4). Peak exercise tolerance (ie, VO_2peak_) or respiratory exchange ratio during graded exercise testing did not change with in the intervention (Table 4).

### 3.5 Quality of life

The mean total score on the MNLWHF decreased by an average of 15 points following RET + protein (Table 4). Of note, the physical domain total decreased and emotional and social domains tended to decrease following RET + protein (Table 5). The largest decrease (*P* = .06) in the physical domain appeared to be in walking or climbing stairs (Table 4).

**TABLE 5:** Effect of 12 weeks of RET + protein on quality of life

MLWHF Domain	Pretraining	Posttraining	*P* value
Physical			
Rest during day	2.8 (2.6); 4 [0‐5]	1.8 (2.5); 0 [0‐4.5]	.19
Walk or climb stairs	3.2 (2.0); 4 [1–5]	1.8 (2.1); 1 [0‐4]	.06
House or yard work	2.6 (2.1); 3 [0.5‐4.5]	1.6 (2.3); 0 [0‐4]	.10
Going places difficult	2.0(2.0); 1 [0.5‐4]	1.8 (2.2); 1 [0‐4]	.32
Sleep well difficult	1.4 (2.2); 0 [0‐3.5]	1.0 (2.2); 0 [0‐2.5]	.32
Things with friends	2.4 (1.8); 2 [1–4]	1.4 (2.2); 0 [0‐3.5]	.10
Short of breath	2.0 (2.0); 1 [0.5‐5]	1.0 (1.7); 0 [0–2.5]	.06
Tired fatigued	2.8 (2.3); 3 [0.5–5]	1.8 (2.5); 0 [0–4.5]	.10
Domain total	19.2 (16); 18 [4‐35]	12.2 (17); 1 [0.5‐29.5]	.04
Emotional			
Burden family	1.6 (2.3); 0 [0–4]	1.4 (2.2); 0 [0‐3.5]	.37
Loss self‐control	1.8 (2.2); 1 [0–4]	1.2 (1.8); 0 [0–3]	.08
Worry	2.4 (1.9); 3 [0.5–4]	1.4 (2.2); 0 [0–3.5]	.10
Difficult Concentrate	1.8 (2.2); 1 [0–4]	1.6 (2.3); 0 [0‐4]	.32
Depressed	1.6 (2.1); 1 [0–3.5]	1.2 (2.2); 0 [0–3]	.16
Domain total	9.2 (10); 4 [1.5‐19.5]	6.8 (11); 0 [0‐17]	.07
Social			
Earn living difficult	3.0 (2.3); 4 [0.5–5]	2.0 (2.7); 0 [0‐5]	.18
Recreation difficult	2.8 (1.9); 3 [1–4.5]	1.8 (2.5); 0 [0–4.5]	.18
Sex difficult	1.8 (2.0); 2 [0–3.5]	1.0 (2.2); 0 [0–2.5]	.16
Costing money	2.8 (2.6); 0 [0‐4.0]	2.2 (2.2); 3 [0–3.5]	.18
Domain total	10.4 (8.0); 12 [2.5‐17.5]	7.0 (8.8); 3 [0–16]	.11
Overall total	43.6 (40) 33 [10.5–82]	27.8 (40); 4.0 [0.5–67]	.04

*Note*: Values are reported mean (SD) and median [IQR]. Wilcoxon Rank Sum.

## 4 DISCUSSION

Data from this pilot study suggest that 12 weeks of resistance exercise plus 42 g/day of whey protein supplementation safely increases muscle strength, improves quality of life, and tends to increase muscle mass in late adolescents and young adults with BTHS. This is the first study to demonstrate an intervention that increases both muscle strength and improves quality of life in individuals with BTHS. Findings from this study, combined with data from our previous study,^7^ suggest that while resistance training improves skeletal muscle strength and quality of life, supplemental protein might be necessary to improve muscle mass and quality of life in late adolescents/young adults with BTHS. Clearly, larger studies are needed to confirm these findings and to clarify the mechanism(s) through which resistance training and protein supplementation improves muscle strength, muscle mass, and quality of life in BTHS.

Our laboratory previously reported the safety and efficacy of a 12‐week RET program in (n = 3) participants with BTHS.^7^ This pilot study found that while resistance training improved muscle strength, critically it did not increase in muscle mass. Although this initial study was underpowered, we hypothesized that the lack of substantial increase in muscle mass might have been in part due to the alterations in leucine turnover and/or plasma amino acid concentrations in individuals with BTHS,^10^, ^11^ and that providing supplemental whey protein with resistance training might increase muscle mass along with improving muscle strength. However, despite adding protein supplementation with resistance exercise training, lean muscle mass only tended to increase with the intervention. These data however are limited in that participant numbers were very small and were not powered a priori to detect differences in body composition. Larger, adequately powered studies are needed to fully assess the effect and mechanisms associated with RET plus protein supplementation on lean muscle mass and strength in BTHS.

The findings of improved muscle strength with resistance exercise and supplemental protein in the current study are consistent with current literature on the effects of resistance training and protein supplementation in healthy individuals without BTHS, although the increase of 1.04 kg in lean mass in our study appears to be approximately half of increases seen in healthy adults.^16^, ^17^, ^18^ In addition, increases in muscle strength following resistance training *without* protein supplementation in individuals with mitochondrial myopathy^19^, ^20^ were also similar to those with BTHS (~average increase in lower extremity strength: mitochondrial myopathy: 20% vs BTHS: 16%). Studies have found that either resistance training alone^20^, ^21^, ^22^ or protein supplementation alone^23^, ^24^ increased skeletal muscle strength and/or mass in populations that share similar characteristics as BTHS (eg, chronic heart failure, mitochondrial myopathy), we are aware of only one study comparing resistance training with and without protein supplementation among patients with chronic heart failure.^25^ However, this study only used 10 g/day of protein, ~25% of that provided in our intervention, and it is possible that this amount may be insufficient to increase muscle mass even in healthy populations.

Reassuringly, we found that the intervention did not increase markers of cardiac muscle damage or heart failure. Additionally, we found that the intervention did not change plasma markers of renal function (ie, creatinine clearance). Moreover, the intervention in the current study did not change left ventricular systolic function but reduced both systolic and diastolic blood pressure in the participants with BTHS. The blood pressure lowering effect with resistance training is consistent with other studies in healthy individuals.^26^ Blood pressure is frequently depressed in BTHS^27^ likely due to a combination of lower systolic function and afterload‐reducing medications and therefore blood pressure during a program of resistance training in patients with BTHS should be monitored closely. Overall, despite the modest reduction in blood pressure, findings suggest that resistance training with 42 g daily protein supplementation is safe and improves muscle strength in adolescents/young adults with BTHS.

Our previous pilot study examining resistance training alone did not find an improvement in quality of life however was not powered to do so. In contrast, we found improved quality of life following resistance training plus protein supplementation. The mean overall 15‐point decrease (ie, improvement) on the Minnesota Living with Hearth Failure questionnaire in BTHS is consistent with the reported effects of exercise training and protein supplementation on quality of life in patients with non‐BTHS related heart failure. Two studies in patients with chronic systolic heart failure examining resistance exercise training alone on quality of life reported that a program of 8‐12 weeks resulted in a mean decrease of −14^28^ and − 8.^29^ Similarly, Wu et al and Rozentryt et al found improvement on MLWHF with a decrease of −12 and −10, respectively^24^, ^30^ with protein/amino acid supplementation in conjunction with outpatient exercise therapy. Importantly, resistance exercise plus protein supplementation improved the physical domain of the MLWHF questionnaire indicating that the intervention improved qualtity of life associated with physical functioning; an area very important to those with BTHS.^9^

Our study has a number of limitations. Our findings are limited by the pilot nature of the study, the small number of participants, and that we did not perform a statistical correction for multiple comparisons and therefore future larger studies are warranted to fully elucidate the impact of this resistance training and protein supplementation in individuals with BTHS. Further, we are not able to directly assess the relative contributions of RET and/or protein supplementation on muscle size/strength. Future cross‐over study designs should be considered to determine the independent effects of RET and protein supplementation in populations with limited number of participants such as BTHS. All of our participants ingested 42 g/day of the whey protein isolate; however, the participants may have received greater benefit from individualised protein amounts based on body weight. Due to technical difficulties, air displacement plethymosgraphy was used to determine pre‐post resistance training body composition vs dual x‐ray absorptiometry for n = 4 participants however this method appears to be rigorous and reliable, providing values that are consistent with DXA.^31^ Our participants were taking beta blockers and other cardiac medications at the time of our study which could have impacted the cardiac function and exercise tolerance results. However, our previous work has shown that there are no differences in heart rate or contractility during graded exercise testing on patients on and off beta‐blocker therapy.^6^ Larger studies examining the effect of RET and protein supplementation on cardiac and skeletal muscle outcomes might elucidate the effects of medications on outcomes.

In conclusion, 12 weeks of RET with 42 g/day whey protein supplementation was safe, improved muscle strength and quality of life and tended to increase muscle mass in late adolescents and young adults with BTHS. Future larger studies are needed to confirm these findings and examine the mechanism(s) through which each of these interventions benefits strength and body composition changes in people living with BTHS.

## CONFLICT OF INTEREST

All authors declare that they have no conflict of interest.

## AUTHOR CONTRIBUTIONS

W. Todd Cade, Kathryn L. Bohnert, Barry J. Byrne, Carolyn Taylor, and Dominic N. Reeds designed the study. W. Todd Cade, Kathryn L. Bohnert, Adam J. Bittel, Lisa de las Fuentes, and Dominic N. Reeds conducted the study. W. Todd Cade, Kathryn L. Bohnert, Barry J. Byrne, and Dominic N. Reeds interpreted the data. Kathryn L. Bohnert, Grace Ditzenberger, and W. Todd Cade wrote the manuscript. All authors edited the manuscript.

## DETAILS OF ETHICS APPROVAL

Studies were approved by the Human Research Protection Office at Washington University in St. Louis.

## PATIENT CONSENT STATEMENT

All minor participants provided assent and all participants and/or parents provided written informed consent.

## References

[1] Clarke SL , Bowron A , Gonzalez IL , et al. Barth syndrome. Orphanet J Rare Dis. 2013;8:23. 23398819 10.1186/1750-1172-8-23 PMC3583704

[2] McKenzie M , Lazarou M , Thorburn DR , Ryan MT . Mitochondrial respiratory chain supercomplexes are destabilized in Barth syndrome patients. J Mol Biol. 2006;361:462‐469. 16857210 10.1016/j.jmb.2006.06.057

[3] Wang G , McCain ML , Yang L , et al. Modeling the mitochondrial cardiomyopathy of Barth syndrome with induced pluripotent stem cell and heart‐on‐chip technologies. Nat Med. 2014;20:616‐623. 24813252 10.1038/nm.3545 PMC4172922

[4] Cade WT , Bohnert KL , Peterson LR , et al. Blunted fat oxidation upon submaximal exercise is partially compensated by enhanced glucose metabolism in children, adolescents and young adults with Barth syndrome. J Inherit Metab Dis. 2019a;42(3):480‐493. 30924938 10.1002/jimd.12094 PMC6483838

[5] Cade WT , Laforest R , Bohnert KL , et al. Myocardial glucose and fatty acid metabolism is altered and associated with lower cardiac function in young adults with Barth syndrome. J Nucl Cardiol. 2019b. 10.1007/s12350-019-01933-3. Online ahead of print. PMC7205570 31705425

[6] Spencer CT , Byrne BJ , Bryant RM , et al. Impaired cardiac reserve and severely diminished skeletal muscle O(2) utilization mediate exercise intolerance in Barth syndrome. Am J Physiol Heart Circ Physiol. 2011;301:H2122‐H2129. 21873497 10.1152/ajpheart.00479.2010

[7] Bittel AJ , Bohnert KL , Reeds DN , et al. Reduced muscle strength in Barth syndrome may be improved by resistance exercise training: a pilot study. JIMD Rep. 2018;41:63‐72. 29654548 10.1007/8904_2018_102 PMC6122057

[8] Hornby B , McClellan R , Buckley L , Carson K , Gooding T , Vernon HJ . Functional exercise capacity, strength, balance and motion reaction time in Barth syndrome. Orphanet J Rare Dis. 2019;14:37. 30744648 10.1186/s13023-019-1006-8 PMC6371525

[9] Food and Drug Administration . The Voice of the Patient: Barth Syndrome. A Report on the Externally‐Led Patient‐Focused Drug Development Meeting. Maryland, USA: FDA, White Oak; 2018.

[10] Cade WT , Spencer CT , Reeds DN , et al. Substrate metabolism during basal and hyperinsulinemic conditions in adolescents and young‐adults with Barth syndrome. J Inherit Metab Dis. 2013;36:91‐101. 22580961 10.1007/s10545-012-9486-x PMC3608431

[11] Vernon HJ , Sandlers Y , McClellan R , Kelley RI . Clinical laboratory studies in Barth syndrome. Mol Genet Metab. 2014;112:143‐147. 24751896 10.1016/j.ymgme.2014.03.007

[12] Bashir A , Bohnert KL , Reeds DN , et al. Impaired cardiac and skeletal muscle bioenergetics in children, adolescents and young adults with Barth syndrome. Physiol Rep. 2017;5(3):e13130. 28196853 10.14814/phy2.13130 PMC5309577

[13] Rector TS , Cohn JN . Assessment of patient outcome with the Minnesota Living with Heart Failure questionnaire: reliability and validity during a randomized, double‐blind, placebo‐controlled trial of pimobendan. Pimobendan Multicenter Research Group. Am Heart J. 1992;124(4):1017‐1025. 10.1016/0002-8703(92)90986-6 1529875

[14] American College of Sports Medicine . American College of Sports Medicine (2000) ACSM's guidelines for exercise testing and prescription. Lippincott, Williams & Wilkins, Baltimore. 2000.

[15] Deutz NE , Bauer JM , Barazzoni R , et al. Protein intake and exercise for optimal muscle function with aging: recommendations from the ESPEN expert group. Clin Nutr. 2014;33:929‐936. 24814383 10.1016/j.clnu.2014.04.007 PMC4208946

[16] Cermak NM , Res PT , de Groot LC , Saris WH , van Loon LJ . Protein supplementation augments the adaptive response of skeletal muscle to resistance‐type exercise training: a meta‐analysis. Am J Clin Nutr. 2012;96:1454‐1464. 23134885 10.3945/ajcn.112.037556

[17] Miller PE , Alexander DD , Perez V . Effects of whey protein and resistance exercise on body composition: a meta‐analysis of randomized controlled trials. J Am Coll Nutr. 2014;33:163‐175. 24724774 10.1080/07315724.2013.875365

[18] Morton RW , Murphy KT , McKellar SR , et al. A systematic review, meta‐analysis and meta‐regression of the effect of protein supplementation on resistance training‐induced gains in muscle mass and strength in healthy adults. Br J Sports Med. 2018;52:376‐384. 28698222 10.1136/bjsports-2017-097608 PMC5867436

[19] Cejudo P , Bautista J , Montemayor T , et al. Exercise training in mitochondrial myopathy: a randomized controlled trial. Muscle Nerve. 2005;32:342‐350. 15962332 10.1002/mus.20368

[20] Murphy JL , Blakely EL , Schaefer AM , et al. Resistance training in patients with single, large‐scale deletions of mitochondrial DNA. Brain. 2008;131:2832‐2840. 18984605 10.1093/brain/awn252

[21] Giuliano C , Karahalios A , Neil C , Allen J , Levinger I . The effects of resistance training on muscle strength, quality of life and aerobic capacity in patients with chronic heart failure ‐ a meta‐analysis. Int J Cardiol. 2017;227:413‐423. 27843045 10.1016/j.ijcard.2016.11.023

[22] Selig SE , Carey MF , Menzies DG , et al. Moderate‐intensity resistance exercise training in patients with chronic heart failure improves strength, endurance, heart rate variability, and forearm blood flow. J Card Fail. 2004;10:21‐30. 14966771 10.1016/s1071-9164(03)00583-9

[23] Nichols S , McGregor G , Al‐Mohammad A , Ali AN , Tew G , O'Doherty AF . The effect of protein and essential amino acid supplementation on muscle strength and performance in patients with chronic heart failure: a systematic review. Eur J Nutr. 2020;59:1785‐1801. 31659450 10.1007/s00394-019-02108-z PMC7351803

[24] Rozentryt P , von Haehling S , Lainscak M , et al. The effects of a high‐caloric protein‐rich oral nutritional supplement in patients with chronic heart failure and cachexia on quality of life, body composition, and inflammation markers: a randomized, double‐blind pilot study. J Cachexia Sarcopenia Muscle. 2010;1:35‐42. 21475692 10.1007/s13539-010-0008-0 PMC3060643

[25] Pineda‐Juarez JA , Sanchez‐Ortiz NA , Castillo‐Martinez L , et al. Changes in body composition in heart failure patients after a resistance exercise program and branched chain amino acid supplementation. Clin Nutr. 2016;35:41‐47. 25726428 10.1016/j.clnu.2015.02.004

[26] Zhang Y , Qi L , Xu L , et al. Effects of exercise modalities on central hemodynamics, arterial stiffness and cardiac function in cardiovascular disease: systematic review and meta‐analysis of randomized controlled trials. PLoS One. 2018;13:e0200829. 30036390 10.1371/journal.pone.0200829 PMC6056055

[27] Spencer CT , Bryant RM , Day J , et al. Cardiac and clinical phenotype in Barth syndrome. Pediatrics. 2006;118:e337‐e346. 16847078 10.1542/peds.2005-2667

[28] Tyni‐Lenne R , Dencker K , Gordon A , Jansson E , Sylven C . Comprehensive local muscle training increases aerobic working capacity and quality of life and decreases neurohormonal activation in patients with chronic heart failure. Eur J Heart Fail. 2001;3:47‐52. 11163735 10.1016/s1388-9842(00)00087-8

[29] Nolte K , Herrmann‐Lingen C , Wachter R , et al. Effects of exercise training on different quality of life dimensions in heart failure with preserved ejection fraction: the ex‐DHF‐P trial. Eur J Prev Cardiol. 2015;22:582‐593. 24627449 10.1177/2047487314526071

[30] Wu C , Kato TS , Ji R , et al. Supplementation of l‐Alanyl‐l‐glutamine and fish oil improves body composition and quality of life in patients with chronic heart failure. Circ Heart Fail. 2015;8:1077‐1087. 26269566 10.1161/CIRCHEARTFAILURE.115.002073 PMC4802502

[31] Fields DA , Higgins PB , Radley D . Air‐displacement plethysmography: here to stay. Curr Opin Clin Nutr Metab Care. 2005;8:624‐629. 16205463 10.1097/01.mco.0000171127.44525.07

